# Longitudinal Response to Epipharyngeal Abrasive Therapy (EAT) in Chronic Nasopharyngitis With Outcome Redefinition and Stratified Analysis by Tissue Type and Baseline Severity

**DOI:** 10.7759/cureus.106426

**Published:** 2026-04-04

**Authors:** Ito Hirobumi

**Affiliations:** 1 Otolaryngology, Ito ENT Clinic, Funabashi, JPN

**Keywords:** bleeding score, chronic epipharyngitis(nasopharyngitis), eat score, edematous type, epipharyngeal abrasive therapy (eat), longitudinal analysis, macroscopic tissue types, outcome definition, proliferative/hypertrophic type, stratified analysis

## Abstract

Objective

This study aimed to evaluate longitudinal changes in nasopharyngeal findings in patients undergoing epipharyngeal abrasive therapy (EAT) and to examine differences in treatment responsiveness according to macroscopic tissue type and baseline severity. In addition, outcome definitions were reconstructed using reproducible criteria based on EAT and bleeding scores, and factors associated with complete resolution were investigated.

Methods

A total of 546 patients undergoing EAT with nasopharyngeal evaluations from baseline up to 12 months were retrospectively analyzed. Inflammation and bleeding were quantified using EAT and bleeding scores. Longitudinal trends (0-12 months) were assessed using mixed-effects models with Restricted Maximum Likelihood (REML) and Holm correction for multiple comparisons. Primary analyses focused on patients with complete data at 0-3 months (n=238), stratified by macroscopic tissue type (proliferative/hypertrophic vs edematous) and baseline severity (EAT score ≥20 vs <20), using Generalized Estimating Equations (GEE). Complete and incomplete resolutions were compared using Welch’s t-test and χ² tests.

Results

Both EAT and bleeding scores decreased significantly from 1 month onward, with improvements maintained up to 12 months. Stratified analyses suggested differential improvement patterns by tissue type and baseline severity, with edematous tissue and higher baseline scores demonstrating faster initial improvement. Complete resolution (n=76) was associated with lower baseline EAT scores, longer treatment duration, higher number of EAT sessions, and higher prevalence of edematous tissue compared to incomplete resolution (n=470). Redefining outcome criteria increased the number of complete resolution cases from 55 in the previous report to 76. Multivariate logistic regression analysis identified age, tissue type, and baseline severity as independent factors associated with complete resolution.

Conclusions

EAT leads to early and sustained improvement in inflammation and bleeding in chronic nasopharyngitis. Stratification by tissue type and baseline severity provides clinically relevant insights for predicting treatment response. Outcome re-definition enhances reproducibility and may inform treatment planning and follow-up strategies.

## Introduction

Chronic nasopharyngitis presents a wide range of symptoms, including local inflammatory symptoms such as postnasal drip, sore throat, pharyngeal discomfort, hoarseness, and nasal congestion, as well as autoimmune-related symptoms such as IgA nephropathy, palmoplantar pustulosis, and atopic dermatitis [1,2], and neuroendocrine symptoms such as dizziness, chronic fatigue, irritable bowel syndrome, and depressive symptoms [3]. Epipharyngeal Abrasive Therapy (EAT) is a treatment technique for chronic nasopharyngitis that has been developed in Japan [4]; it involves abrasion of the nasopharyngeal mucosa under endoscopic guidance with the aim of improving local findings [5]. The nasopharynx may function not merely as a site of infection defence but as a central organ involved in the formation and maintenance of immune memory [6,7].

Hotta et al. proposed a hypothesis regarding the mechanism of action of EAT, suggesting that its therapeutic effects are due to anti-inflammatory actions resulting from the suppression of inflammatory cytokines, the resolution of submucosal edema, and the removal of inflammatory factors and cellular debris through bloodletting, thereby reporting the theoretical basis for the therapeutic efficacy of EAT [8]. Furthermore, stimulation of the nasopharynx by EAT may activate the Central Autonomic Network (CAN), potentially exerting regulatory effects on the autonomic, endocrine, and immune systems [9,10]; however, this mechanism remains hypothetical and has not been directly demonstrated in the present study. In contrast, the observed clinical responses were not uniform, varying among patients, and treatment efficacy differed according to baseline symptom severity [8].

Ohno et al. retrospectively evaluated 73 patients with chronic nasopharyngitis who underwent EAT using 1% zinc chloride solution. Patients received ten EAT sessions, and improvements were assessed using a four-point scale for subjective symptoms and endoscopic evaluation of redness, swelling, and postnasal drip. The study reported a symptom improvement rate of 79.5% and a local finding improvement rate of 87.7%, with a significant correlation between symptom and local finding improvement, confirming EAT as an effective treatment for chronic nasopharyngitis [11].

Despite these findings, the nasopharynx is anatomically challenging to observe, and symptoms are often nonspecific, which can lead to subjective assessment of diagnosis and treatment efficacy. In clinical practice, mucosal appearance and the degree of bleeding during abrasion are commonly used as indicators of treatment response. However, treatment responsiveness may differ across patients, necessitating evaluation of optimal follow-up intervals and treatment duration [3]. Systematic longitudinal assessment of nasopharyngeal mucosal inflammation and vulnerability remains limited. Furthermore, factors associated with complete resolution have not been sufficiently investigated, and evidence to support clinical decision-making remains limited.

This study was conducted using the same dataset of 546 patients as the first report [12]. In this study, the group classification based primarily on clinical cure decisions used in the first report was revised, and outcome definitions and classifications were reconstructed based on EAT and bleeding scores. Longitudinal and stratified analyses were then performed to re-evaluate the time course and characteristics of treatment response. Even when using the same dataset as the first report, differences in definitions and classifications can change the number of patients considered cured and alter the interpretation of group comparisons; therefore, re-analysis using reproducible criteria has significant clinical relevance.

In this study, 546 patients who underwent EAT were classified into two macroscopic tissue types, proliferative/hypertrophic and edematous, for analysis. Chronic nasopharyngitis includes variants such as the nasopharyngeal roof type, characterized by localized edema and swelling near the nasopharyngeal roof, and the Tornwaldt type, which is characterized by crust formation near the Tornwaldt cyst region. In this study, these subtypes were treated as a subtype of the edematous category and were included in the edematous group for analysis [12]. Longitudinal changes following treatment initiation were evaluated using quantitative EAT scores for inflammation and bleeding scores to reflect mucosal friability. Furthermore, stratified analyses were performed according to macroscopic tissue type (proliferative/hypertrophic vs edematous) and baseline severity (baseline EAT score) to assess differences in treatment responsiveness.

Factors associated with final outcomes (complete vs incomplete resolution) were also explored to provide clinically relevant insights for evaluating EAT efficacy and optimizing treatment strategies. However, standardized and longitudinally evaluated endoscopic outcome measures reflecting inflammatory activity and mucosal fragility in chronic nasopharyngitis remain insufficiently established. Therefore, this study aimed to evaluate longitudinal responses to EAT using reproducible endoscopic criteria and to clarify treatment response patterns through stratified analyses.

## Materials and methods

Study design and participants

This study was a retrospective single-center observational study of patients who underwent epipharyngeal abrasive therapy (EAT) and whose nasopharyngeal findings were recorded longitudinally. The study was conducted at Ito ENT Clinic, and the study period was from August 1, 2018, to August 31, 2019. The study population consisted of patients with available baseline data, including at least the initial EAT score and bleeding score at the first visit. Consecutive patients who met the diagnostic criteria for chronic nasopharyngitis during the study period were included. As no universally validated diagnostic criteria for chronic nasopharyngitis are currently available, the diagnosis was based on predefined clinical and endoscopic criteria consistently applied within the institution. The inclusion criteria were the persistence of nasopharyngeal inflammatory symptoms for more than one month after onset, the absence of other structural diseases of the nasopharynx, and the presence of nasopharyngeal inflammatory findings confirmed by endoscopic examination.

Outcome measures

The primary outcome was the EAT score (inflammatory findings), and the secondary outcome was the bleeding score (mucosal friability) [12]. Systemic symptoms related to chronic nasopharyngitis, rated on a 0-5 Numerical Rating Scale (NRS), totalled to a maximum of 50 points. The bleeding score assessed the two-dimensional extent of bleeding areas on the nasopharyngeal mucosa induced by EAT. The posterior nasopharyngeal wall was divided into four quadrants (upper-right, upper-left, lower-right, lower-left) relative to the central fossa of the pharyngeal tonsil, and scores were assigned from 0 to 3 according to the bleeding extent. Both scores were recorded at baseline and at each monthly follow-up from 1 to 12 months. Detailed definitions of the scores and outcome criteria are provided in Table 1.

**Table 1:** EAT score, bleeding score, and outcome definitions. EAT Score (Self-reported symptoms): The EAT score is a composite measure of self-reported symptoms related to chronic nasopharyngitis. It evaluates symptoms in 10 categories, including postnasal drip, throat discomfort, throat pain, and others. Each category is scored on a 0-5 scale (NRS, Numerical Rating Scale), with higher scores indicating greater severity. The total score ranges from 0 to 50, where higher scores indicate more severe symptoms. Bleeding Score (Susceptibility to bleeding): The bleeding score evaluates the extent of mucosal bleeding in the nasopharynx induced by epipharyngeal abrasive therapy (EAT). The distribution of bleeding was assessed in four regions centered on the pharyngeal tonsillar fossa: upper right, upper left, lower right, and lower left. Based on the extent of bleeding, a score ranging from 0 to 3 was assigned. A score of 0 indicates no bleeding or only minimal bleeding after abrasion. A score of 1 indicates mild bleeding involving one region. A score of 2 indicates moderate bleeding involving two regions. A score of 3 indicates severe bleeding involving three to four regions. Treatment outcomes were classified into four categories based on the EAT score and bleeding score. Complete resolution was defined as cases in which the EAT score was ≤10 and the bleeding score was 0. Partial resolution was defined as cases in which either the EAT score was ≤10 or the bleeding score was 0. Treatment discontinuation was defined as cases that did not meet the criteria for complete or partial resolution. Indeterminate referred to cases in which only baseline data were recorded at the initial visit and no subsequent evaluations were available. EAT: epipharyngeal abrasive therapy;  NRS: numerical rating scale.

Item	Definition/assessment method
EAT score (subjective symptoms)	Subjective symptoms related to chronic nasopharyngitis were classified into 10 domains Postnasal drip–related symptoms (postnasal drip, etc.), nasopharyngeal irritation–related symptoms (abnormal throat sensation, headache, shoulder stiffness, etc.), nasal-related symptoms (rhinorrhea, nasal obstruction, nasal irritation symptoms, etc.), Eustachian tube–related symptoms (aural fullness, tinnitus, etc.), vertigo-related symptoms, voice-related symptoms (hoarseness, voice disorders, etc.), laryngeal irritation–related symptoms (cough, sore throat, etc.), sleep apnea–related symptoms (snoring, daytime sleepiness, etc.), gastrointestinal symptoms (gastric acid reflux, dyspepsia, etc.), autonomic nervous system–related symptoms (chronic fatigue, depressive symptoms, etc.). Each domain was rated on a 6-point NRS (0–5) and summed.
NRS 6-point scale (each EAT domain)	0: No symptoms 1: Slight/rarely noticeable 2: Mild/occasionally noticeable 3: Constantly noticeable 4: Marked/almost always noticeable but tolerable 5: Very severe and intolerable
Bleeding score (bleeding tendency)	The two-dimensional extent of mucosal bleeding induced by EAT was evaluated. The posterior wall of the nasopharynx was divided into four quadrants (upper right, upper left, lower right, lower left) centered on the pharyngeal tonsil fossa. The score was determined by the number of involved regions.
Bleeding score criteria	0: No bleeding (no bleeding or only minimal oozing after abrasion) 1: Mild (1 quadrant) 2: Moderate (2 quadrants) 3: Severe (3–4 quadrants)
Outcome definition (this study)	Patients were classified based on EAT score outcome and bleeding score outcome.

Stratification factors

To clarify differences in treatment response, stratified analyses were conducted based on macroscopic tissue type (proliferative/hypertrophic vs edematous) and baseline severity (baseline EAT score ≥20 as high vs <20 as low). Stratification was used to identify differences in treatment response and to provide guidance for individualized treatment strategies. Macroscopic findings of the nasopharyngeal mucosa in patients undergoing epipharyngeal abrasive therapy (EAT) were classified into two tissue types: the proliferative/hypertrophic type and the edematous type.

Proliferative/Hypertrophic Type

This type is characterized by mucosal thickening, granular surface changes, and irregular mucosal architecture of the nasopharyngeal mucosa. The mucosa appears relatively thick and uneven, reflecting chronic inflammatory changes associated with fibrosis and tissue proliferation. These findings are considered to represent a more chronic and structurally fixed inflammatory state.

Edematous Type

**Figure 1:**
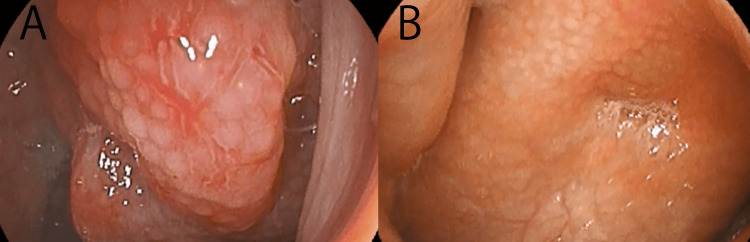
Macroscopic tissue types of chronic nasopharyngitis. Macroscopic findings of the nasopharyngeal mucosa in patients undergoing epipharyngeal abrasive therapy (EAT) were classified into two tissue types: A. the proliferative/hypertrophic type and B. the edematous type. Endoscopic images represent typical examples of each tissue type. All images are original photographs obtained at Ito ENT Clinic and have not been previously published.

This type is characterized primarily by mucosal edema and swelling, with the mucosa appearing soft and bulging. Increased vascular visibility and mucosal friability during abrasion are frequently observed. These findings are considered to reflect relatively reversible inflammatory changes associated with submucosal edema and microcirculatory disturbance. Chronic nasopharyngitis may also present with morphological variants such as the nasopharyngeal roof type, characterized by localized edema and swelling near the nasopharyngeal roof, and the Tornwaldt type, characterized by crust formation around the Tornwaldt cyst region. In the present study, these variants were treated as subtypes of the edematous type and were included in the edematous group for analysis (Figure 1). 

Outcome definitions (differences from the first report)

In the first report, patients were classified as cured based on clinical cure decisions [12]. In this study, complete resolution was defined as cases in which both the EAT and bleeding score outcomes reached the cure threshold. All other cases were classified as incomplete resolution (partial resolution, discontinued, or not assessable). The thresholds used to define complete resolution were selected based on clinical interpretability and consistency with routine endoscopic assessment; however, they have not been externally validated. Partial resolution included cases where either the EAT or bleeding score outcome reached cure. Cases in which neither score reached cure by the end of observation, or in which treatment was interrupted for any reason after the first month, were considered discontinued. Cases with only baseline evaluation were classified as not assessable. This redefinition of outcomes allowed cases that did not meet the original cure criteria or were not recorded in the first report [12] to be reclassified as complete resolution, increasing the number of complete resolution cases from 55 in the first report to 76 in this study.

Statistical analysis (software and methods)

Statistical analyses were performed using EZR (version 1.70; Saitama Medical Center, Jichi Medical University, Saitama, Japan), GraphPad Prism (version 10.6.1; GraphPad Software, Boston, MA, USA), and Python (version 3.11.7; Python Software Foundation, Wilmington, DE, USA) with the statsmodels package (version 0.14.4). All statistical tests were two-sided, and a p-value <0.05 was considered statistically significant.

Patient characteristics were summarized using descriptive statistics. Changes between baseline and month 3 within each subgroup were evaluated using paired t-tests. Longitudinal changes over 0-12 months (supplementary analysis including missing data) were analyzed using a mixed-effects model with patient ID as a random intercept, estimated by restricted maximum likelihood (REML), to test for time effects. Time was treated as a categorical variable consisting of baseline and each month (months 1-12). Comparisons between each time point and baseline were adjusted for multiple testing using the Holm method. All p-values were adjusted for multiple comparisons using the Holm method. For the primary group × time analysis over 0-3 months, the analysis was restricted to patients with complete data at all time points (baseline, month 1, month 2, and month 3). To account for within-subject correlation arising from repeated measurements, generalized estimating equations (GEE) were applied (working correlation structure: exchangeable; distribution: Gaussian; robust standard errors).

In the primary 0-3 month analysis, a complete-case analysis approach was adopted, including only patients with complete data at baseline, month 1, month 2, and month 3. A complete-case analysis was adopted to ensure consistency in longitudinal comparisons, although this approach may introduce selection bias. Time over 0-3 months was treated as a continuous variable representing a linear trend, and differences in the slope (rate of improvement) between groups were evaluated using the group × time interaction term. Comparisons between the complete cure group and the non-complete cure group were performed using Welch’s t-test for continuous variables and the chi-square test (χ² test) for categorical variables. Additionally, multivariate logistic regression analysis was performed to identify independent factors associated with complete resolution. The model included age, sex, tissue type, baseline EAT score, and baseline bleeding score. Adjusted odds ratios (ORs) with 95% confidence intervals (CIs) were calculated.

Ethical considerations

This study adhered to the Declaration of Helsinki. Patients were provided with detailed information regarding the study objectives, the scope of information to be used, and the research process, and were given the option to decline participation. Informed consent was obtained both in writing and orally. Data were anonymized and managed with access restrictions to protect participant privacy. Study results were reported without any identifiable information. The study protocol was approved by the Chiba Prefecture Health Insurance Association Ethics Committee (approval number 20200625006), and the research was conducted in accordance with the committee’s guidelines to respect participant rights.

## Results

Patient characteristics

The baseline characteristics of the 546 patients included in this study are summarized in Table 2. The most common chief complaint was postnasal drip. The distributions of age, sex, duration of illness, macroscopic tissue type, baseline EAT score, and baseline bleeding score are also summarized.

**Table 2:** Patient background (n=546). This table presents the demographic and clinical background of the 546 patients who were included in the study. The values include age, sex, disease duration, endoscopic tissue type, baseline EAT score, baseline bleeding score, and chief complaints. The data is presented in the following formats: Age: Mean ± SD and Median [IQR]; Sex: Number (%) of male and female patients; Disease duration: Mean ± SD and Median [IQR] in months; Endoscopic tissue type: The distribution of patients based on the type of tissue observed endoscopically (hypertrophic/proliferative or edematous); Baseline EAT score: Mean ± SD and Median [IQR] at the time of the initial examination; Baseline bleeding score: Mean ± SD and Median [IQR] of bleeding at the time of the initial examination; Chief complaints (Top 10): The most common complaints reported by the patients, along with their respective percentages.

Item	Value
Age (years)	50.2 ± 16.3
Sex	
Male	136 (24.9%)
Female	410 (75.1%)
Disease duration (months)	8.0 [1.0–36.0]
Tissue type	
Hypertrophic/proliferative	303 (55.5%)
Edematous	243 (44.5%)
Baseline EAT score	19.0 [13.0–25.0]
Baseline bleeding score	3.0 [2.0–3.0]
Chief complaint	
Postnasal drip	243 (44.5%)
Throat discomfort	61 (11.2%)
Throat pain	40 (7.3%)
Hoarseness	37 (6.8%)
Nasal obstruction	28 (5.1%)
Cough	18 (3.3%)
Headache	12 (2.2%)
Rhinorrhea	11 (2.0%)
Dizziness	7 (1.3%)
Chronic fatigue	5 (0.9%)

Longitudinal changes in EAT score (0-12 months)

**Figure 2:**
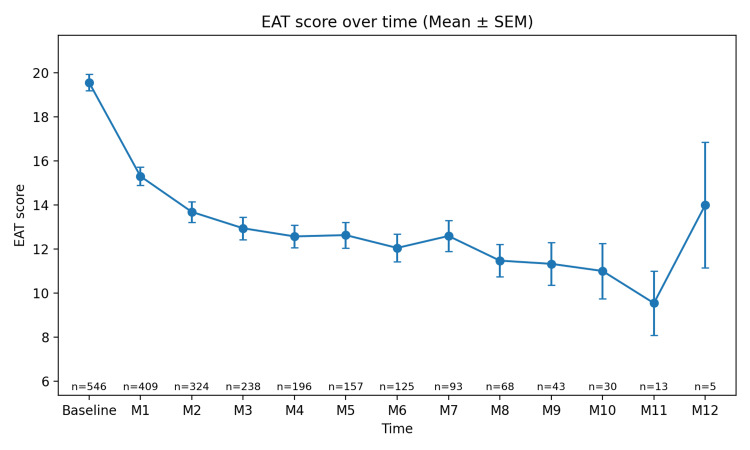
EAT score over time (Mean ± SEM) The plot illustrates the change in EAT (Epipharyngeal Abrasive Therapy) score over time. The EAT score represents the severity of symptoms associated with chronic epipharyngitis, measured at baseline and at each subsequent month (M1 to M12). The data points represent the mean EAT score at each time point, and the error bars represent the Standard Error of the Mean (SEM), indicating the variability or precision of the estimate at each time point. The sample size decreases over time because not all patients completed the full 12-month follow-up period. Some patients discontinued treatment or had incomplete follow-up data. Time points: Data are presented at baseline and at months 1 (M1), 2 (M2), 3 (M3), …, and 12 (M12); Sample size (n): The number of patients included at each time point is shown below each data point (e.g., baseline n=546; M1 n=409); Statistical presentation: Values are expressed as Mean ± Standard Error of the Mean (SEM), which reflects the precision of the estimated mean; EAT score: The EAT score represents the severity of chronic epipharyngitis, with higher scores indicating greater disease severity.

Longitudinal changes in the EAT score were analyzed using a mixed-effects model estimated by restricted maximum likelihood (REML). The main effect of time was statistically significant (χ²(12)=624.29, p=6.87×10⁻¹²⁶). The mean EAT score at the initial visit was 19.57±0.37 points (n=546). A significant decrease was observed from the first month after treatment initiation (e.g., difference at 1 month −4.49 points [95% CI −5.13, −3.85], Holm-adjusted p=9.99×10⁻⁴³), and the declining trend was generally maintained until month 11. At month 12, the number of observations was small (n=5), and the mean value fluctuated, appearing relatively higher. However, according to the estimates obtained from the mixed-effects model, the EAT score at month 12 remained significantly lower than the baseline value (difference at 12 months −8.90 points [−13.25, −4.55], Holm-adjusted p=6.14×10⁻⁵). As shown in Figure 2, these findings confirm the longitudinal decrease in the EAT score over time.

Longitudinal changes in bleeding score (0-12 months)

**Figure 3:**
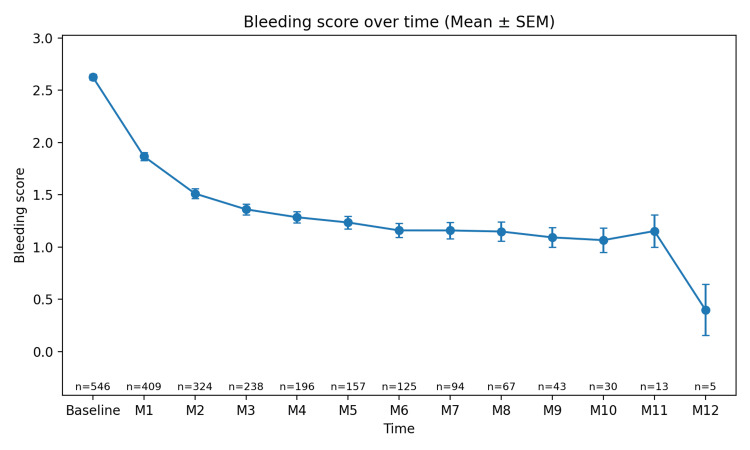
Bleeding score over time (Mean ± SEM). The plot illustrates the change in bleeding score over time. The Bleeding score measures the extent of bleeding induced by the Epipharyngeal Abrasive Therapy (EAT) at each time point, from baseline (pre-treatment) to each month from M1 to M12. The data points represent the mean bleeding score at each time point, and the error bars represent the Standard Error of the Mean (SEM), indicating the variability or precision of the estimate at each time point. The sample size decreases over time because not all patients completed the full 12-month follow-up period. Some patients discontinued treatment or had incomplete follow-up data. Time points: data are presented at baseline (pre-treatment) and at months 1 (M1), 2 (M2), 3 (M3), …, and 12 (M12); sample size (n): the number of patients at each time point is indicated below each data point (e.g., baseline n=546; M1 n=409); data presentation: values are expressed as mean ± standard error of the mean (SEM); bleeding score: the bleeding score ranges from 0 to 3, with higher values indicating more extensive bleeding.

Longitudinal changes in the bleeding score were analyzed using a mixed-effects model estimated by restricted maximum likelihood (REML). The main effect of time was statistically significant (χ²(12)=1499.72, p=4.36×10⁻³¹⁴). The mean bleeding score at the initial visit was 2.626±0.027 (n=546). A significant decrease was observed from the first month after treatment initiation (e.g., difference at 1 month −0.775 [95% CI −0.856, −0.693], Holm-adjusted p=1.71×10⁻⁷⁶), and values remained lower than baseline at all time points up to month 12 (difference at 12 months −2.330 [−2.899, −1.761], Holm-adjusted p=9.90×10⁻¹⁶). As shown in Figure 3, these results confirm the longitudinal decline in the bleeding score over time.

Stratified analysis (primary 0-3-month analysis: group × time)

**Figure 4:**
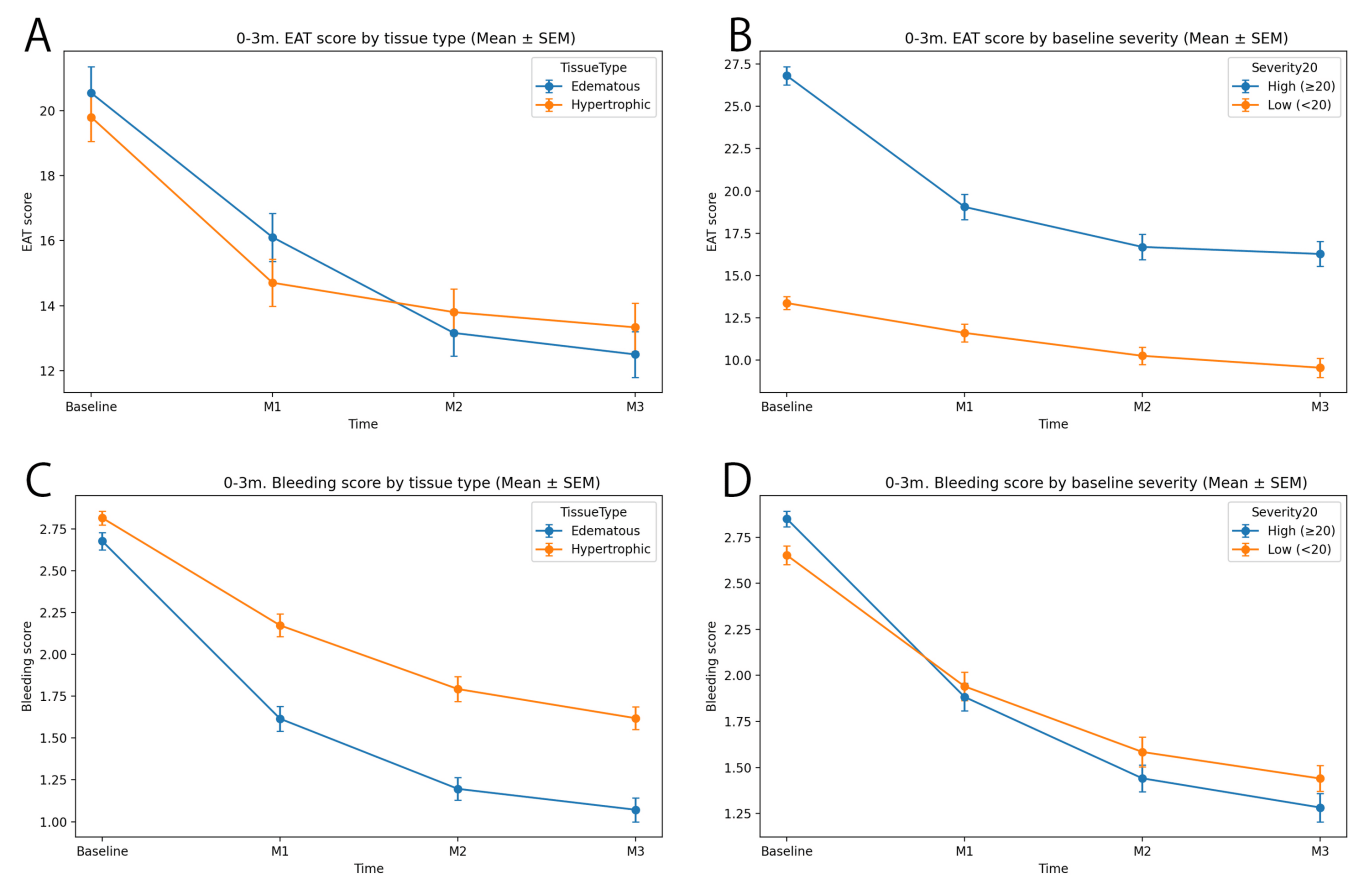
Stratified analysis (primary 0–3-month analysis: group × time). A: This plot compares the change in EAT score over time (from baseline to M3) between two groups based on tissue type: edematous (blue line) and hypertrophic (orange line). The EAT score represents the severity of symptoms associated with chronic nasopharyngitis. The data points show the mean EAT score at each time point, and the error bars represent the standard error of the mean (SEM). B: This plot compares the EAT score change over time (from baseline to M3) between patients grouped by their baseline severity score. The high baseline severity group (EAT score ≥ 20, blue line) is compared with the low baseline severity group (EAT score < 20, orange line). The data points show the mean EAT score at each time point, with error bars representing the SEM. C: This plot compares the change in Bleeding score over time (from baseline to M3) between two groups based on tissue type: edematous (blue line) and hypertrophic (orange line). The Bleeding score measures the extent of bleeding induced by EAT in the nasopharyngeal mucosa. The data points show the mean Bleeding score, and the error bars represent the standard error of the mean (SEM). D: This plot compares the Bleeding score change over time (from baseline to M3) between patients grouped by their baseline severity (EAT score ≥ 20 vs. EAT score < 20). The High severity group (EAT score ≥ 20, blue line) is compared with the Low Severity group (EAT score < 20, orange line). The data points show the mean Bleeding score, and the error bars represent the SEM. Time points: baseline (pre-treatment), M1, M2, and M3; statistical methods: Mean ± SEM: the mean score is plotted at each time point with the SEM indicating variability; A higher EAT score indicates more severe symptoms of chronic nasopharyngitis; A higher bleeding score indicates a wider area of bleeding.

A total of 238 patients had complete data at all time points from baseline to month 3 (baseline, month 1, month 2, and month 3). The results are presented in Table 3 and Figure 4. Table 3 shows the mean scores and the number of patients at baseline and at month 3 for each subgroup. Changes between baseline and month 3 within each subgroup were evaluated using paired t-tests, and the results are presented in Table 3. EAT scores decreased in all subgroups. In particular, patients with higher baseline EAT scores (≥20) showed greater reductions compared with those with lower baseline scores (<20). In the analysis by tissue type, decreases in EAT scores were observed in both the edematous type and the hypertrophic/proliferative type. The magnitude of improvement was substantial, with mean EAT scores decreasing by approximately 6-10 points from baseline to month 3 across the analyzed subgroups.

**Table 3:** Changes in EAT score and bleeding score from baseline to month 3 according to tissue type and baseline severity. Values are presented as mean ± standard deviation (SD). The analysis included patients with complete data at all time points from baseline to month 3 (n=238). EAT score and bleeding score were evaluated at baseline (pre-treatment) and at month 3. Subgroup analyses were performed according to endoscopic tissue type (edematous type vs hypertrophic/proliferative type) and baseline disease severity (baseline EAT score ≥ 20 vs < 20). Statistical comparisons between baseline and month 3 within each subgroup were performed using paired t-tests, which assess whether the mean difference between two repeated measurements within the same individuals differs from zero. The t-value represents the standardized difference between the paired measurements relative to their variability. The p-value indicates the probability that the observed difference occurred by chance under the null hypothesis of no change between baseline and month 3. A p-value < 0.001 indicates a statistically significant reduction in scores after treatment. EAT score represents the severity of chronic epipharyngitis, with higher values indicating more severe inflammation. Bleeding score is an ordinal scale ranging from 0 to 3, reflecting the extent of bleeding observed during EAT.

Outcome	Grouping factor	Subgroup	n (0–3m complete)	Baseline mean ± SD	Month 3, mean ± SD	t statistic	p-value
EAT score	Tissue type	Edematous	112	20.54 ± 8.64	12.50 ± 7.41	10.76	<0.001
EAT score	Tissue type	Hypertrophic/proliferative	126	19.80 ± 8.33	13.33 ± 8.31	9.21	<0.001
EAT score	Baseline severity	≥20	120	26.82 ± 5.93	16.27 ± 8.08	13.75	<0.001
EAT score	Baseline severity	<20	118	13.37 ± 4.19	9.55 ± 6.07	7.3	<0.001
Bleeding score	Tissue type	Edematous	112	2.68 ± 0.56	1.07 ± 0.76	18.04	<0.001
Bleeding score	Tissue type	Hypertrophic/proliferative	126	2.82 ± 0.46	1.62 ± 0.76	16.21	<0.001
Bleeding score	Baseline severity	≥20	120	2.85 ± 0.46	1.28 ± 0.84	18.41	<0.001
Bleeding score	Baseline severity	<20	118	2.65 ± 0.55	1.44 ± 0.76	15.56	<0.001

Longitudinal changes over time were further analyzed using generalized estimating equations (GEE) to account for within-subject correlation in repeated measurements, and the results are shown in Figure 4.

In the tissue type-stratified analysis of EAT scores, the EAT score decreased significantly over time (linear time effect, p=1.72×10⁻²⁸). Improvement was observed in both the edematous group (blue line) and the proliferative/hypertrophic group (orange line); however, in the edematous group, the EAT score improved and then appeared to stabilize thereafter. This improvement tended to be sustained over the long term. Clinically, these findings may be interpreted as indicating that, in patients with the edematous type, symptoms are alleviated early after treatment initiation and the therapeutic effect can be perceived relatively quickly. In contrast, patients with the proliferative/hypertrophic type showed a slower pace of improvement than those with the edematous type, and the degree of improvement after treatment was more gradual. This suggests that patients with the hypertrophic type may require long-term treatment and continuous evaluation, and that more time may be needed before treatment effects become apparent. In addition, differences were observed in the pattern of EAT score improvement between the edematous and proliferative/hypertrophic groups, and the group × time interaction (difference in slopes) was also significant (p=0.0401). This interaction indicated that improvement may be steeper in patients with the edematous type, whereas the pace of improvement may be slower in patients with the proliferative/hypertrophic type (Figure 4A).

Furthermore, in the analysis stratified by baseline severity of the EAT score (baseline EAT score ≥20 vs <20), a significant time effect was observed for the EAT score (p=2.54×10⁻⁴⁴). Patients in the high-score group (≥20) at the initial visit showed marked improvement after treatment, with a large magnitude of change. In particular, among patients in the high-score group (blue line), the score decreased substantially by the first month after treatment initiation, indicating a rapid improvement. In contrast, patients in the low-score group (<20) showed a smaller magnitude of improvement, with a more gradual decrease in scores. These results suggest that the treatment may be more effective in patients with more severe disease and highlight the importance of early intervention in severe cases. The group × time interaction was also significant (p=1.94×10⁻¹²), indicating that patients with higher baseline EAT scores exhibited more pronounced improvement after treatment compared with those with lower baseline scores (Figure 4B).

Next, tissue-type-stratified analysis of the bleeding score was performed. The bleeding score also decreased significantly over time (linear time effect, p=2.05×10⁻⁷⁴). Patients with the edematous type (blue line) showed a clear improvement early after treatment initiation, and by the first month, the bleeding score had significantly decreased compared with the baseline value. These findings indicate that improvement after treatment appeared earlier in patients with the edematous type than in those with the proliferative/hypertrophic type. In contrast, patients with the proliferative/hypertrophic type (orange line) showed more gradual improvement after treatment initiation, and the decrease in bleeding score was slower. These results demonstrate that the rate of improvement in the bleeding score depends on tissue type. Early follow-up after treatment initiation may therefore allow early confirmation of symptom improvement in patients with the edematous type. A significant group × time interaction was also observed for the bleeding score (p=9.61×10⁻⁴), indicating that the treatment response differed over time between patients with the edematous type and those with the proliferative/hypertrophic type (Figure 4C).

In addition, analysis of the bleeding score stratified by baseline severity showed that the bleeding score decreased significantly over time after treatment initiation (p=1.82×10⁻⁷⁵). Significant reductions were observed in both the high-severity group (EAT score ≥20) and the low-severity group (EAT score <20), suggesting that treatment was effective in improving the bleeding score regardless of baseline severity. However, no significant difference between groups was observed (p=0.5504), indicating that there was no difference in baseline bleeding scores between the high- and low-severity groups. This suggests that treatment responsiveness may not depend on baseline severity at the initial visit.

The group × time interaction for the bleeding score was significant (p=0.00262), confirming that the degree of improvement after treatment differed according to severity. In the high-severity group, improvement in the bleeding score following treatment was more pronounced, whereas the magnitude of improvement in the low-severity group was relatively smaller. These findings indicate that the therapeutic effect may be more evident in patients with more severe disease (Figure 4D).

Comparison between complete resolution and incomplete resolution

In the prognostic analysis, patients were divided into a complete resolution group (n=76) and an incomplete resolution group (n=470). Statistical analyses were performed using Welch’s t-test for continuous variables and the χ² test for categorical variables. The results showed that the complete resolution group exhibited the following characteristics compared with the incomplete resolution group.

First, the complete resolution group was significantly older than the incomplete resolution group (58.67±15.24 years vs 48.86±16.34 years, p=1.20×10⁻⁶). This finding suggests that older patients may have a greater likelihood of responding favorably to treatment.

Second, the baseline EAT score at the initial visit was significantly lower in the complete resolution group (17.38±8.66 vs 19.92±8.62, p=0.0196). This indicates that patients with lower levels of inflammation at baseline may have a higher probability of achieving complete resolution.

Furthermore, the duration until the final outcome assessment was significantly longer in the complete resolution group (3.94±2.21 months vs 2.38±2.75 months, p=1.08×10⁻⁶). Similarly, the number of EAT sessions until the final assessment was significantly greater in the complete resolution group (6.64±2.79 vs 4.43±2.49, p=3.57×10⁻⁹). These results suggest that achieving complete resolution may require a longer treatment period and a greater number of treatment sessions. In addition, comparison of macroscopic tissue types showed that the proportion of the edematous type was significantly higher in the complete resolution group (67.1% vs 40.9%, p=3.3×10⁻⁵). Patients with the edematous type demonstrated better treatment responsiveness and were more likely to achieve complete resolution compared with those with the proliferative/hypertrophic type. Taken together, these findings indicate that characteristics associated with patients achieving complete resolution include older age, lower baseline EAT scores, longer treatment duration, a greater number of EAT sessions, and the presence of the edematous tissue type (Table 4). 

**Table 4:** Comparison of clinical characteristics between patients with complete resolution and those with incomplete resolution. Values are presented as mean ± standard deviation or n (%). Continuous variables were compared using the independent-samples t-test, and categorical variables were analyzed using the chi-square test. Effect sizes are reported as Cohen’s d for continuous variables and odds ratios (OR) for categorical variables. Notably, the edematous tissue type was more frequently observed in patients who achieved complete resolution than in those with incomplete resolution (OR=2.95), suggesting that patients with the edematous type may have a higher likelihood of achieving complete resolution following EAT.

Variable	Complete resolution (n=76)	Incomplete resolution (n=470)	Test statistic	P-value	Effect size
Age (years)	58.67 ± 15.24	48.86 ± 16.34	t = 5.16	<0.001	d = 0.61
Disease duration (months)	51.80 ± 85.20	41.20 ± 74.07	t = 1.02	0.308	d = 0.14
Baseline EAT score	17.38 ± 8.66	19.92 ± 8.62	t = -2.37	0.019	d = -0.29
Baseline bleeding score	2.66 ± 0.62	2.62 ± 0.64	t = 0.47	0.637	d = 0.06
Time to EAT ≤10 or censor (months)	2.29 ± 1.70	1.79 ± 2.18	t = 2.28	0.024	d = 0.24
Time to bleeding =0 or censor (months)	3.47 ± 2.05	2.43 ± 2.71	t = 3.91	<0.001	d = 0.40
Time to final judgment (months)	4.00 ± 2.19	2.55 ± 2.81	t = 5.12	<0.001	d = 0.53
Total EAT procedures	30.43 ± 16.05	12.17 ± 11.58	t = 9.53	<0.001	d = 1.49
Sex (female)	62 (81.6%)	348 (74.0%)	χ² = 1.60	0.205	OR = 1.55
Tissue type (edematous)	51 (67.1%)	192 (40.9%)	χ² = 17.21	<0.001	OR = 2.95

In addition, multivariate logistic regression analysis demonstrated that age (OR 1.03, 95% CI 1.01-1.05, p=0.001), edematous tissue type (OR 2.20, 95% CI 1.23-3.92, p=0.008), baseline EAT score (OR 0.97, 95% CI 0.94-1.00, p=0.040), and baseline bleeding score (OR 1.64, 95% CI 1.05-2.57, p=0.029) were independently associated with complete resolution. Sex was not significantly associated with the outcome (Table 5). 

**Table 5:** Multivariate logistic regression analysis for factors associated with complete resolution. Multivariate logistic regression analysis was performed using complete resolution (binary outcome: 0 no, 1=yes) as the dependent variable. Independent variables included age (continuous), sex (male/female), tissue type (edematous vs hypertrophic/fibrotic), baseline EAT score, and baseline bleeding score. The analysis identified age, tissue type, baseline EAT score, and baseline bleeding score as independent predictors of complete resolution. Older age and edematous tissue type were associated with a higher likelihood of complete resolution, whereas higher baseline EAT scores were associated with a lower likelihood of resolution. In contrast, higher baseline bleeding scores were associated with a greater likelihood of resolution. Sex was not significantly associated with the outcome. Odds ratios (ORs) represent the strength and direction of association between each variable and complete resolution. The 95% confidence interval (CI) indicates the range within which the true effect size is likely to lie, and statistical significance was determined by p-values, with p < 0.05 considered significant.

Variable	Odds ratio (OR)	95% confidence interval	p-value
Age (per year increase)	1.03	1.01–1.05	0.001
Sex (female vs male)	1.10	0.57–2.11	0.780
Tissue type (edematous vs hypertrophic/proliferative)	2.20	1.23–3.92	0.008
Baseline EAT score (per point increase)	0.97	0.94–1.00	0.040
Baseline bleeding score (per point increase)	1.64	1.05–2.57	0.029

Sensitivity analyses were performed to assess the robustness of the findings by comparing results from the complete-case analysis (patients with complete data at baseline through month 3) and the overall dataset analysis, including patients with missing values. The direction and magnitude of the observed effects were generally consistent between the two approaches. In particular, the time-dependent improvement in EAT scores and bleeding scores, as well as the differences according to tissue type and baseline severity, remained statistically significant in both analyses. These findings indicate that the main results are robust and are unlikely to be substantially influenced by missing data or differences in analytical approach.

The results of this study indicate that age, baseline EAT score, treatment duration, number of treatment sessions, and tissue type are important factors associated with achieving complete resolution. In particular, older patients were more likely to achieve complete resolution. However, this finding requires further investigation to clarify its relationship with treatment responsiveness and age-related changes in immune function. Patients with lower baseline EAT scores tended to show earlier treatment responses, suggesting that a lower degree of initial inflammation may facilitate a more rapid therapeutic effect.

Future studies should investigate the mechanisms underlying treatment responsiveness in patients with lower EAT scores in order to better understand how these patients respond to EAT. In addition, the association between a greater number of treatment sessions and a longer treatment duration with complete resolution suggests that more intensive and sustained treatment may have a substantial impact on treatment outcomes. Extending the treatment period may increase the likelihood of achieving complete resolution; however, optimization of treatment plans should also consider treatment quality and patient burden. The difference in treatment response between the edematous and proliferative/hypertrophic tissue types also represents an important finding. Patients with the edematous type showed earlier responses to treatment, suggesting the potential for a targeted treatment strategy in future therapeutic approaches. In contrast, patients with the proliferative/hypertrophic type may require longer-term treatment and continuous follow-up to achieve optimal outcomes.

## Discussion

This study demonstrated that EAT scores and bleeding scores were associated with consistent improvement from the early phase following EAT, as shown in longitudinal data from a large cohort. Improvements were observed from the first month in the 0-12-month analysis, suggesting that EAT may be associated with relatively rapid changes in nasopharyngeal mucosal findings, and that assessment at 1 month may be clinically useful for early evaluation. Improvements were maintained up to 12 months, and treatment response patterns appeared to vary according to baseline severity and tissue type, suggesting that these factors may be associated with differences in EAT responsiveness and may be useful for guiding follow-up intervals (early evaluation for rapid responders and continued treatment guidance for slower responders).

Primary 0-3-month repeated-measures analysis suggested that the slope of improvement differed according to tissue type (proliferative/hypertrophic vs edematous) and baseline severity (baseline EAT score ≥20). Stratification by baseline severity showed not only significant group differences but also a significant group × time interaction, indicating that higher-severity patients may experience steeper early improvement gradients. Clinically, this suggests that even severe cases may respond early, which is useful for patient counselling regarding early treatment effects and motivation for continued therapy.

Evaluation was performed according to the prospective assessment methods established by the EAT Committee of the Japanese Society of Oral and Pharyngeal Science, including endoscopic assessment (color, swelling, mucus attachment/postnasal drip, and bleeding during abrasion scored on a three-point scale) and subjective symptom assessment (improvement of postnasal drip, sore throat, cough, and other symptoms scored on a four-point scale). Additional cases were incorporated from previous studies [11] for re-evaluation [13]. The results demonstrated that EAT produced marked improvements in both endoscopic findings and subjective symptoms in patients with chronic nasopharyngitis, with bleeding showing particularly high rates of improvement and, in most patients, resolution or substantial reduction after treatment. However, it was reported that while the extent of bleeding is useful for evaluating baseline severity, further consideration is required before including it as a primary measure for treatment efficacy [13]. In the present study, stratification by bleeding score did not always reveal clear differences between severity groups, although the group × time interaction was significant. This indicates that inflammatory findings and mucosal friability may not improve at the same rate, suggesting that improvement in symptoms and findings cannot be fully represented by a single measure. Therefore, concurrent assessment using both EAT scores and bleeding scores is important for evaluating treatment response.

Stratification by tissue type revealed clearer differences in bleeding scores, with edematous patients tending to show higher values. Edematous changes are often associated with submucosal congestion, microcirculatory disturbances, and epithelial barrier vulnerability, which may explain the increased bleeding tendency during abrasion. EAT has been reported to reduce the expression of inflammatory cytokines in chronic nasopharyngitis [8,14]. Dysfunctional ciliated epithelium is removed during EAT, allowing replacement by squamous epithelial cells and restoration of barrier function in the nasopharyngeal mucosa [14].

Chronic inflammatory diseases typically progress from an acute/activity phase, characterized by edema and cellular infiltration, to a chronic/fibrotic phase [15]. Chronic nasopharyngitis may similarly be divided into an activity phase dominated by edema (edematous type) and a fibrotic phase (proliferative/hypertrophic type). Edematous patients are prone to bleeding and may exhibit rapid treatment responses, whereas proliferative/hypertrophic patients show delayed responses and less reversible tissue changes, requiring prolonged therapy. Observed differences in EAT score improvement by tissue type suggest that the primary site of treatment response may vary depending on whether the pathology is predominantly proliferative/hypertrophic or edematous. Future studies should investigate inter-rater reliability for tissue classification and incorporate objective endoscopic scoring to achieve more precise stratification.

Patients achieving complete resolution were older and had lower baseline EAT scores compared to those with incomplete resolution. Lower baseline scores likely reflect milder or more reversible pathology at the time of intervention, consistent with achieving resolution. The finding that older patients achieved complete resolution more frequently may seem counterintuitive, but it could reflect confounding factors such as treatment adherence, comorbidities, or lifestyle. For example, older patients may maintain more consistent follow-up, ensuring a sufficient number of EAT sessions. Indeed, the complete resolution group had longer follow-up and more EAT sessions, suggesting that adequate treatment intensity and duration are important for achieving full resolution. This indicates that while EAT may induce rapid short-term improvements, achieving complete resolution requires sustained therapy. Edematous tissue type was more prevalent in the complete resolution group, consistent with a higher proportion of reversible changes that respond well to intervention. These associations should not be interpreted as causal but should be evaluated using multivariate analyses, adjusting for confounders, such as logistic regression. In the present study, multivariate logistic regression analysis confirmed that some of these factors were independently associated with complete resolution. These findings support the results of the univariate analysis and suggest that the observed associations cannot be fully explained solely by confounding factors.

The study results support the clinical validity of EAT as a treatment for chronic nasopharyngitis, demonstrating improvement in both EAT scores (inflammatory findings) and bleeding scores (mucosal friability) [11,13,16]. While treatment response varies among individuals, pathology significantly influences responsiveness. Based on autonomic pathophysiology and differential response to EAT, the author proposed the EAT Field Theory and the EAT Reflex Adjustment 3-Phase Model [10]. Differences in response according to tissue type and immune reactivity suggest that treatment effects may vary by pathological phenotype. Responses are continuous but may include mixed types. Future studies are needed to clarify which phenotypes respond most strongly and the characteristic response patterns, including underlying physiological and neuroimmunological mechanisms. These mechanistic interpretations remain hypothetical and require further validation in future studies.

The early improvement in EAT and bleeding scores observed from 1 month suggests that these measures may serve as early indicators of treatment response. Improvements were maintained up to 12 months, and response patterns differed according to baseline severity and tissue type. Chronic nasopharyngitis treated with EAT may thus be classified into distinct trajectories, such as early responders and late responders, based on resolution patterns.

This study, while using the same dataset as the first report [12], differs in that outcome definitions and classifications were updated. In the first report, clinical operational resolution (cure decision) was used, whereas in this study, complete resolution was defined using reproducible criteria based on EAT and bleeding score outcomes. This allowed cases previously unclassified or not meeting prior operational criteria to be reclassified as complete resolution, increasing the number of complete resolution cases from 55 in the first report to 76 in the current study. The significance of this second report lies in improved reproducibility, appropriate handling of longitudinal data including missing values, and demonstration of differential responses by tissue type and baseline severity, providing guidance for optimal assessment timing and patient counselling.

This study has several limitations. First, its retrospective single-center design may limit generalizability and introduce potential selection bias. Second, the lack of a control group prevents exclusion of natural course or concomitant treatment effects. Third, missing data over 0-12 months may have biased analyses toward patients with continued follow-up. Fourth, restriction of the primary 0-3-month analysis to patients with complete data may have further introduced bias toward a population with high adherence. Fifth, clinical assessment of EAT and bleeding scores may include inter-rater variability. Outcome redefinition using the same dataset may have introduced bias toward an apparent increase in complete resolution, potentially inflating treatment effects. In addition, redefining outcomes using the same dataset may have introduced outcome redefinition bias. Although multivariate analysis was performed in the revised analysis, earlier unadjusted findings may have been influenced by residual confounding. 

Future studies should integrate symptom indices (e.g., VAS), quality-of-life measures, objective endoscopic findings (black spots, granular changes, whitening) [17], and inflammatory markers [18] to systematically evaluate the relationship between findings and patient outcomes. In addition, external validation in multicenter prospective studies is warranted to confirm the generalizability of these findings. The present findings should be interpreted with caution, given the observational design, outcome redefinition, and potential selection bias due to complete-case analysis.

## Conclusions

EAT was associated with early and sustained improvement in both symptom and bleeding scores in patients with chronic nasopharyngitis. Treatment responses appeared to vary according to endoscopic tissue type and baseline severity, suggesting that disease stratification may be important for optimizing EAT evaluation and follow-up. By redefining outcomes with reproducible endoscopic criteria and applying longitudinal stratified analyses in a large cohort, this second report may provide a clinically relevant framework for interpreting treatment responses to EAT. Multivariate analysis suggested that age, tissue type, and baseline severity were independently associated with complete resolution. As this is an observational study, causality cannot be inferred.
